# Are we teaching health science students in the United States what they need to know about death and dying coping strategies?

**DOI:** 10.3352/jeehp.2021.18.29

**Published:** 2021-11-11

**Authors:** Randy D. Case, Erica Judie, Tammy Kurszewski, Wenica Brodie, Pollyann Bethel

**Affiliations:** 1 Department of Respiratory Care, Midwestern State University, Wichita Falls, TX, USA; 2 Department of Respiratory Therapy, Children’s Medical Center Dallas, Dallas, TX, USA; 3 Department of Respiratory Therapy, Tallahassee Memorial Healthcare, Tallahassee, FL, USA; Hallym University, Korea

**Keywords:** Curriculum, Death, Educational status, Perception, Students

## Abstract

**Purpose:**

This investigation aimed to answer the following questions: are health science students provided with death and dying education before attending clinical rotations, and if so, do the students receiving this type of education perceive it as effective?

**Methods:**

In this descriptive cross-sectional survey, 96 Midwestern State University health science students were surveyed to determine the percentage of students who had received death and dying education before clinical rotations, as well as the students’ perception of educational effectiveness for those who had received end-of-life training. A self-report questionnaire presented nursing, radiologic sciences, and respiratory care students with a series of questions pertaining to the education they had received concerning the death and dying process of patients.

**Results:**

Of the 93 students who had already started their clinical rotations, 55 stated they had not received death and dying education before starting clinical courses. Of the 38 who had received death and dying education, only 17 students believed the training was effective.

**Conclusion:**

It is imperative that health science educational programs implement death and dying education and training into the curriculum, and that criteria for evaluating effectiveness be an essential part of death and dying education and training in order to ensure effectiveness.

## Introduction

### Background/rationale

Situations involving the death and dying process are common events within the healthcare environment. Although frequently observed, many healthcare professionals still find it difficult to cope with the death and dying process. Grief associated with the death of a patient can often result in both psychological and behavioral reactions [1]. Psychological responses such as guilt, sadness, depression, anxiety, and fatigue are often observed in those treating patients during the death and dying process [1]. Behavioral reactions can also be observed in healthcare professionals who are consistently exposed to experiences associated with death. Some of these reactions can include health-compromising activities, self-destructive actions, and risk-taking behaviors [1]. Similarly, health science students play a role in comparable experiences associated with death during their clinical rotations. However, these events are often more emotionally taxing for students than for experienced healthcare professionals. This is often due to students’ lack of clinical experience and the deficiency of education about end-of-life care. Proper education and training relating to end-of-life care have the potential to reduce the emotional responses of those entering the healthcare environment. This type of specific education provides students with improved coping mechanisms, communication skills, and self-care strategies. Death and dying education also provides students with the understanding that death is a common occurrence in the hospital setting.

Death and dying education can be instructed in numerous ways. Traditional lecture-based content about end-of-life care can be provided to students. This method allows the didactic components of the content to be presented to the students. Simulation is another method to demonstrate the death and dying process to students. This format provides experiential learning related to the death of a patient. Simulation experiences allow students the opportunity to be subjected to the death and dying process in a controlled, learning environment. However, it remains to be determined if health science students are instructed during their educational training to manage the emotional and behavioral impacts related to the death of a patient. This article explored research about the importance and effectiveness of death and dying education for healthcare students. Some current health science students were also surveyed to determine if they were provided end-of-life training and if so, the effectiveness of the death and dying education they had received before clinical rotations.

### Objectives

This investigation aimed to verify if health science students receive training associated with the death and dying process of patients. The study also sought to determine if students who had end-of-life training believed the education was effective in dealing with the emotional and behavioral responses associated with the death of a patient. The hypotheses of the study were as follows:

#### Hypothesis 1

A larger percentage of surveyed students have received adequate end-of-life training in comparison to those who have not received training.

#### Hypothesis 2

A larger percentage of surveyed students do not feel the end-of-life training they received was effective in comparison to those who feel it was effective.

## Methods

### Ethics statement

Approval from the Institutional Review Board of Midwestern State University was obtained (approval no., 19030608). Informed consent was obtained from the participants. Participation in the study was completely voluntary and an explanation of the survey was provided to each student.

### Study design

This study was a descriptive cross-sectional survey based on a survey questionnaire.

### Setting

The questionnaires were distributed and completed by participants in the Centennial Hall Health Science building on the Midwestern State University in Wichita Falls, Texas after 2 interdisciplinary exercises involving students from the nursing, radiologic sciences, and respiratory programs. In total, 127 students participating in interdisciplinary activities had the opportunity to take part in the study. The data collection period was from January 14 to April 10, 2019. A total of 96 responses to questionnaires were collected and analyzed. The results of 3 participating students were excluded from the study because they had not participated in clinical courses at the time of the survey.

### Participants

A convenience sample of health science students, including those from the nursing, radiologic sciences, and respiratory care programs from Midwestern State University, was recruited for this study. The sample of participating students was limited to those who agreed to complete the survey for the study. No other exclusion criteria were applied.

### Variables

The 7 items of the questionnaire were the variables measured in this study (Supplement 1).

### Data sources/measurement

This study used the Student Views of Death (SVD)–revised questionnaire, which was based on the Physician Views on Death questionnaire. Use of the questionnaire was permitted by the corresponding author of this measurement tool. The SVD questionnaire was developed to discover students’ responses concerning terminally ill patients, and to address students’ preparedness for end-of-life education and patient care. Its content validity was originally assessed by Rio et al. [2] using the Lawshe formula with 14 expert judges. The discriminative capacity of each item was previously calculated using the extreme-groups method. Items were analyzed individually, and responses of agreement were scored in one category, undetermined responses in another, and those of disagreement in yet another. The Cohen’s kappa coefficient was between 0.87 and 1. The survey consisted of 2 demographic questions to ascertain the student’s program of study and to verify that the student had participated in clinical rotations prior to the survey. No other identifying criteria were used. The remaining 6 questions gathered information pertaining to the student’s experience with death and dying in the clinical setting, the preparations given as a part of their preclinical training, and how effective the preparations, if any, were in helping participants deal with the loss of a patient. The questions used in the survey can be seen in Supplement 1.

### Bias

There may have been sampling bias because not all target students participated in the survey.

### Study size

No sample size estimation was done because only voluntarily participating students were included.

### Statistical methods

Descriptive statistics were used to analyze result frequencies from questions 1 through 7.

## Results

### Descriptive data of study participants

No demographic findings were collected from participants.

### Main results

**Fig. 1.**
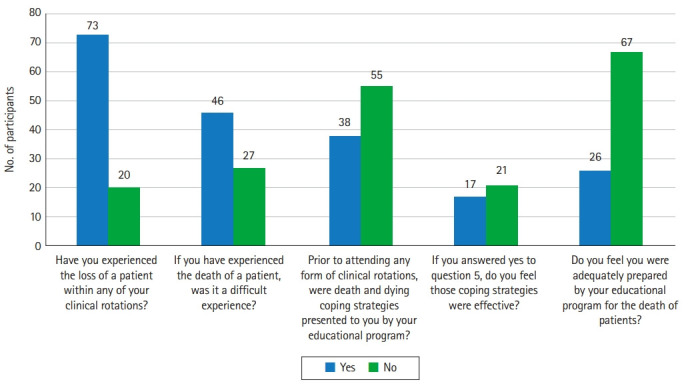
Results of survey questions.

Of the 96 participants, 93 had already begun their clinical rotations. Of those 93 students, the majority had experienced the death of a patient during their clinical rotations, as seen in Fig. 1. Similarly, 63% of those who had experienced the death of a patient felt the experience was difficult to deal with. When asked if death and dying coping strategies were presented by their educational programs before attending any form of clinical rotation, 55 of the 93 students stated that they had not received this type of training or education, as seen in Fig. 1 (Dataset 1). This result refutes the first hypothesis that a larger percentage of students would have received education and training about the death and dying process. Of the 38 students who had received education about coping with death and dying, only 17 stated that the coping strategies they received were effective. This result confirms the second hypothesis that a larger percentage of students would not classify their death and dying education as effective. Sixty-seven of the surveyed students did not believe their educational program adequately prepared them to manage the emotional and behavioral consequences associated with the death of a patient.

Question 8, which provided surveyed students the opportunity to provide specific coping strategies they believed would be beneficial to health science students, found several themes regarding the suggested coping strategies. Twenty-seven of the 93 students surveyed provided similar answers demonstrating the desire to simply talk and discuss the death and dying process. One student stated within the survey, “It would be good to hear how someone in the medical field handles death.” Communication, debriefing, and discussing what happens during and after the death of a patient were prominent themes within the survey. Twenty-one students wanted to know better strategies and effective ways to handle family members of patients who have died. The most recognized theme associated with this question centered on the different types of self-care. Thirty-four students responded to the question by addressing numerous ways to destress and deemphasize the anxiety and tension created by a traumatic event, such as the death of a patient. Whether it be exercise, reading, or getting a massage, the surveyed students were curious as to which form of self-care was the most effective in preventing emotional and behavioral responses to death.

## Discussion

### Interpretation

The above findings suggest that students did not receive adequate training and education on death and dying before entering their clinical rotations. As mentioned above, this study refuted the hypothesis that a larger percentage of students had received end-of-life training and education before starting clinical rotations. This verifies the need for the university to provide death and dying education for its health science students. The study also confirmed the hypothesis that a larger percentage of students would classify the death and dying education they received as not effective. This strengthens the argument that the university needs to provide advanced educational strategies associated with end-of-life care to improve the overall effectiveness of education.

### Comparison with previous studies

Minimal research has been conducted to determine students’ perspectives and coping abilities associated with the death or dying process of patients [3]. A 2019 study by Cerit [4] of 81 first-year nursing students examined the impact of training and education centered on the care provided to a dying patient. Pre- and post-tests utilizing the Death Attitude Profile-Revised survey were given to the students to establish their overall attitude and perceptions concerning the care of dying patients. The students were surveyed before death and dying training and then again after the training. The pre-test surveys found that the majority of students expressed genuine fear, anxiety, and incompetence associated with caring for a dying patient [4]. The post-test results found a significant increase in students’ positive attitudes regarding care for dying patients [4]. However, the question remains: what strategies and educational methods can be utilized to effectively help students with the death and dying process?

Although no specific technique has demonstrated an absolute ability, there are several methods and strategies to effectively convey the knowledge and skills associated with the death and dying process. Developing the necessary communication skills to provide empathy to those who have lost a loved one can be difficult and takes time to cultivate. Therefore, providing training and education to health science students which combines communication and empathy could be quite beneficial. A study by Hawkins and Tredgett [5] found that it was beneficial for medical students to participate in high-fidelity simulation exercises to strengthen communication skills when dealing with a patient in cardiac arrest. Students in that study expressed they gained more knowledge and improved their confidence to a greater extent when using the high-fidelity simulations than when using low-fidelity simulations or lecture-based learning methods. Another valuable strategy in assisting health science students to manage the death of patients is the use of the Death Café model forum. Nelson et al. [6] described the Death Café model as a social setting that simply allowed participants to discuss and engage with each other about the death and dying process. In their study, 24 students from five different health science majors participated in a Death Café event. The participants of that study described the event as valuable and an overall positive experience. The students also expressed that the Death Café experience allowed them to evaluate the death process in a more relaxed and positive environment, which in turn developed a desire “to learn more about issues surrounding death and dying [6].” In turn, the students expressed a better understanding of the dying process and believed they had gained confidence when asked to discuss and interact with those directly affected by the death and dying experience.

Perez-de la Cruz and Ramirez [7] discovered the importance of emotional connection and sensitivity training for health science students. In their research of 548 health science students from diverse professional programs, 82.7% of those surveyed understood that the death and dying process would be frequently seen within their profession. However, 40.4% of those surveyed did not feel they were adequately prepared to cope with the emotional and behavioral effects associated with the death and dying process upon entering their field of practice. Those who did feel prepared for delivering end-of-life care reported that learning about the need for compassion and sensitivity to those experiencing death was the most profound training they received [7]. One aspect that stood out in the study of Perez-de la Cruz and Ramirez [7] was not the method utilized to teach the students, but more the content that was taught. The researchers found it to be more beneficial to implement training modules that focused on appropriate responses and sensitivity to human suffering than on the technical and scientific aspects of death [7].

Although effective communication and a proper understanding of the death and dying process are essential for health science students, the ability to cope with the emotions and feelings associated with a dying patient is also crucial. As previously discussed, one of the most significant themes found in the present study was students’ desire to understand strategies used as self-care mechanisms to combat stress and other emotional responses associated with death and dying. According to Forster and Hafiz [8], several key factors are connected to developing a balanced and effective strategy to cope with the death of patients. These factors include but are not limited to a strong support group of family and friends, spiritual beliefs, acknowledgment of each death versus avoidance, and allowing oneself to experience joy even during difficult times. However, Forster and Hafiz [8] addressed the fact that not all of the mentioned elements are necessary or required to meet an individual’s self-care needs.

Nia et al. [9] explained the importance of practicing healthy eating, drinking, sleeping, and exercise habits when grieving the loss of a patient. In many situations involving direct care for dying patients, health care professionals have relied on unhealthy and potentially dangerous habits such as drugs and alcohol to avoid and escape the emotional responses associated with death. However, these activities and behaviors are extremely hazardous and may result in adverse outcomes. Unfortunately, these basic and essential elements of coping with the death and dying process are often never discussed with health science students [10]. Regardless of the instructional methodology, the introduction and development of death and dying education for health science students must be addressed.

### Limitations

Due to the convenience-based selection of students, the surveys were not equally distributed among various groups of healthcare students. This limitation could be addressed by distributing surveys during activities that provide an equal opportunity for all health science students. In addition, the participants of the survey were students from a single university. It would be beneficial to evaluate different strategies and educational objectives from several universities. Future studies conducted at different universities could result in finding students who are well-equipped and prepared for the death and dying process. Evaluating other universities’ methods of delivering death and dying education could also help determine effective teaching strategies to implement in the health science curriculum.

### Conclusion

According to the findings of this study, a significant number of students within varied health science fields did not feel that they had received adequate preparation for death and dying at the collegiate level. In an effort to reduce the psychological and behavioral responses associated with the death of patients, it is imperative to provide health science students with the fundamental skills of coping and managing the death and dying process by incorporating timely and effective death and dying classes or training in the curriculum.

By doing so, healthcare students may be better equipped and prepared to care for those in the final stages of life while also maintaining their emotional stability and mindset.

## Footnotes

**Data availability**
Data files are available from Harvard Dataverse: https://doi.org/10.7910/DVN/UAYJ3G Dataset 1. Raw data of students’ responses about the death and dying education survey.

## Supplementary materials

Data files are available from Harvard Dataverse: https://doi.org/10.7910/DVN/UAYJ3G

Supplement 1. Survey questionnaire about death and dying education.

Supplement 2. Audio recording of the abstract.
