# Supplementary material for: Are we teaching health science students in the United States what they need to know about death and dying coping strategies?
Source: J Educ Eval Health Prof. 2021 Nov 11;18:29. doi: 10.3352/jeehp.2021.18.29 (PMC8616726; doi:10.3352/jeehp.2021.18.29)
Supplement: Supplementary file 2 — Supplement 1. Survey questionnaire about death and dying education. [file jeehp-18-29-suppl1.docx]

CONSENT FORM *

I hereby give my consent for my participation in the project entitled: The psychological impact of death and dying on health care students. Completion of the survey implies that you have read the information in this form and consent to take part in the research.

I understand that the persons responsible for this project is Randy Case, Wenica Brodie, and Pollyann Bethel, telephone number, 940-337-8737.

These studies are part of a project that has the following objectives:

1.To determine if health science students are educated and trained for the death and dying process prior to attending clinical rotations.

2.To determine if the training they may have received was effective.

   Randy, Wenica, and Pollyann, authorized representatives have *(*1) explained the procedures to be followed and identified those that are experimental.

*No Payment or compensation will be provided for participation in this study. Participation is completely voluntary, and subject can refuse to be involved.*

It has further been explained to me that the total duration of my participation will be 10 minutes, that only Randy Case, Wenica Brodie, and Pollyann Bethel will have access to the records and/or data collection for this study.

Dr. Randy Case, Dr Erica Judie, and Dr. Tammy Kurszewski have agreed to answer any inquiries I may have concerning the procedures and have informed me that I may contact the Midwestern State University Human Subject Review Committee by writing to: Chair, Human Subjects Review Committee, c/o Office of the Provost, Midwestern State University, 3410 Taft Blvd., Wichita Falls, TX 76308, or by calling the Provost at (940) 397-4226.

There are no major risks involved in answering the questionnaire completely and truthfully. However, there is a potential for minimal discomfort or distress in responding to questions about death and dying. If you experience any discomfort or distress due to the survey, you may contact the University Counseling Center to talk with someone (940-397-4618). If this research project causes any physical injury to participants in this project, treatment is not necessarily available at Midwestern State University Student Health Center, nor is there necessarily any insurance carried by the University or its personnel to cover such an injury.  Financial compensation for any such injury must be provided through the participant’s own insurance program. Further information about these matters may be obtained from Chair, Human Subjects Review Committee, c/o Office of the Provost, Midwestern State University, 3410 Taft Blvd., Wichita Falls, TX 76308.

I understand that I may not derive therapeutic treatment from participation in this study.  I understand that I may discontinue my participation in this study at any time without penalty.

Signature of Subject _____________________________________________ Date ___________________

Death and Dying Survey

1. What is your program of study? _____________________________________________
2. Have you participated in clinical rotations for your program of study?
   - Yes
   - No
3. Have you experienced the loss of a patient within any of your clinical rotations?
   - Yes
   - No
4. If you have experienced the death of a patient, was it a difficult experience?
   - Yes
   - No
5. Prior to attending any form of clinical rotations, were death and dying coping strategies presented to you by your educational program?
   - Yes
   - No
6. If you answered yes to question 5, do you feel those coping strategies were effective?
   - Yes
   - No
7. Do you feel you were adequately prepared by your educational program for the death of patients?
   - Yes
   - No
8. Open-ended question: What death and dying coping strategies do you believe should be taught within health science educational programs?

_______________________________________________________________________________________________________________________________________________________________________________________________________________________________________________________________
